# Developing Master Adaptive Learners: Implementation of a Coaching Program in Graduate Medical Education

**DOI:** 10.5811/westjem.2022.12.57636

**Published:** 2023-01-11

**Authors:** Margaret Wolff, Maya Hammoud, Michele Carney

**Affiliations:** * University of Michigan Medical School, Department of Emergency Medicine and Pediatrics, Ann Arbor, Michigan; † University of Michigan Medical School, Department of Obstetrics and Gynecology, Ann Arbor, Michigan

## BACKGROUND

Medical knowledge is expanding exponentially, outpacing the information that can be learned during medical training.^1^ To keep pace with this rapid acceleration of new information, physicians must continually address practice gaps and adapt to novel situations.^2^,^3^ Unfortunately, studies demonstrate physicians frequently perform inaccurate self-assessments and generate learning goals discordant with gaps.^4^ This suboptimal approach to learning is attributed, in part, to medical training not preparing physicians to be effective learners and highlights the need to teach learners to learn.^2^,^5^–^6^ Despite this, there is a paucity of published curricula or programs delineating how to train trainees to learn effectively.

This imperative for continuous learning is palpable in the complex and challenging clinical setting of the emergency department (ED). The acuity, fast pace, and variability in patient presentations require physicians to nimbly adapt to novel challenges.^7^ These same factors that make effective, continuous learning necessary to provide high-quality patient care also make the ED a challenging place to learn.^7^ Therefore, this intervention focused specifically on a novel coaching approach to develop life-long learning skills in pediatric emergency medicine (PEM) fellows at a large academic center.

## OBJECTIVES

Coaching is a collaborative process focused on helping the learner achieve their full potential by improving their self-monitoring, facilitating goal-setting, and providing accountability.^3^ We developed, piloted, and evaluated a novel coaching approach consisting of longitudinal, on-shift, and group coaching designed to facilitate the development of skills, processes, and habits necessary to become career-long, self-directed learners who can adapt. We identified the conceptual framework of the master adaptive learner (MAL) as the basis for our program.^3^ This framework combines principles from quality improvement (plan-do-study-act) and the educational theory of self-regulated learning to describe an approach to learning.^3^ This model describes learners moving through four integrated phases that begin with identifying a knowledge or skills gap (planning phase), engaging in learning to address this gap (learning phase), and assessing learning and receiving feedback (assessing phase), followed by incorporation of the newly learned information (adjusting phase).

Although there are no published curricula to develop MAL skills, various approaches have been proposed in the literature.^3^,^5^,^8^,^9^ These proposals emphasize the central role of educators in facilitating MAL development and creating a supportive learning environment. Specifically, educators must provide teaching that focuses on metacognition, direct observation with frequent feedback, and support for learning plan development.^8^–^10^ Coaching has been suggested as a facilitator of MAL development because of the emphasis on gap identification, goal creation, and personal accountability in coaching.^3^ In addition, the Coalition for Physician Accountability recommends coaching to promote effective lifelong learning across the learner continuum.^5^

## PROGRAM DESIGN

We performed a needs assessment in December 2020–January 2021 of our PEM fellows to identify their current approach to learning and application of MAL skills. One author (MW) performed semi-structured interviews with three recent graduates, four fellows, and one incoming fellow. We performed focus groups with faculty to understand their approach to fellow education, direct observation, feedback, and learning plan development. The fellows lacked a defined approach to learning and pointed to infrequent and poor-quality feedback as barriers to identifying gaps. The faculty cited multiple challenges to providing feedback and acknowledged infrequently identifying actionable steps to improve their performance.

This pre-pilot needs assessment highlighted the need for an approach that included the fellows and the faculty supervising them. Therefore, we developed a three-pronged approach of group, on-shift, and longitudinal coaching (Table 1) to focus on different aspects of MAL development. The group coaching sessions focused on the metacognition of learning using the MAL framework. Faculty and fellows learned alongside each other to share ideas, create shared language, and build community and trust. Initial sessions focused on building coaching skills for the faculty and coachee skills for the fellows, using an in-the-moment coaching approach.^11^ The MAL framework was introduced in the initial session and MAL concepts and skills were layered on in subsequent sessions. On-shift and longitudinal coaching reinforced these concepts and provided fellows opportunities to practice the elements of MAL – specifically reflection, goal-setting, and action plan development. On-shift coaching is a type of performance coaching focused on gap identification, performing informed self-assessment and creating an active plan for change, which are key components of the MAL process. The goal of longitudinal coaching with their fellowship program director was to reflect on on-shift coaching, review written feedback and overall development, and to set goals.

To gain support, we elicited fellow and faculty input early in the development. Prior to implementation, fellows identified a cohort of trusted faculty who were recruited to provide on-shift coaching. Faculty and fellows learned the approach for on-shift coaching during the group coaching sessions and had opportunities to practice via role play. Division leadership approved creating this role for faculty to include in their educators’ portfolios to increase buy-in.

The pilot ran from April 2021–February 2022. Faculty and fellows participated in six group-coaching sessions facilitated by a certified coach. In between the sessions, monthly emails provided fellows and faculty an opportunity to ask questions, provide feedback, and troubleshoot barriers. At monthly division meetings, we provided suggestions for effective feedback to all faculty and highlighted faculty selected by the fellows for their exemplar on-shift feedback and coaching. This served to celebrate successes and raise awareness of the initiative.

The faculty and fellows were surveyed at the end of the pilot regarding two key MAL processes: feedback behaviors and development; and use of learning goals and plans. Using these results, we developed a follow-up interview questionnaire. Using a phenomenological approach, one author (MW) conducted follow-up, semi-structured interviews in February and March 2022 with fellows who participated in the program to explore their experience and the impact of the program on their development of MAL skills. Data was categorized by two authors (MW and MC) using the a priori identified codes of the phases of MAL: planning; learning; assessing; and adjusting. The study was deemed exempt by our institutional review board.

## IMPACT

Table 1 summarizes the results of the pilot survey. The fellows’ experience of the program and their MAL development based on the semi-structured interviews is described below, and representative quotations are shown in Table 2.

### Impact on Approach to Learning

Fellows described how the pilot positively influenced their approach to learning and normalized their development by creating a conversation about learning. The regular intervals of the group and on-shift coaching kept learning at the forefront and provided opportunities to learn from each other. They described feedback evolving from a static conversation to an ongoing conversation throughout shift. The fellows described growth in the planning and assessing phases of MAL, as described below. The learning and adjusting phases were not impacted significantly.

### Impact on Planning

Fellows described being more proactive in identifying gaps and intentionally seeking feedback from multiple sources over the course of the pilot. The fellows described on-shift coaching as crucial to their identification of gaps, reinforcement of positive behaviors, and overall growth as a pediatric emergency physician. Fellows reported trying out methods to identify gaps that their colleagues shared during the group sessions. Fellows differentiated learning tasks for medical knowledge from learning goals focused on procedural skills and non-technical skills such as interprofessional communication or leadership. They described creating intentional learning goals and ongoing learning plans for procedural skills and non-technical skills and not being as specific about medical knowledge.

Prior to starting the pilot, the fellows routinely prioritized gaps necessary for immediate patient care while on shift. The gaps not immediately critical to patient care were often lost by the time they had time to learn. While this remained effortful throughout the pilot, faculty and fellows shared strategies such as keeping a list on their phone, keeping annotated patient logs, and saving patients in a personal follow-up list on the electronic health record for keeping track of the gaps they wanted to address.

The longitudinal sessions prompted the fellows to reflect on their overall performance, develop goals, and revisit prior goals. The sessions also served to validate progress and set expectations for performance.

### Impact on Assessing

The fellows described on-shift coaching to be crucial to the informed self-assessment process. In addition, the fellows described feeling more comfortable trying out new things and being vocal about asking for feedback.

Although this program had success in facilitating MAL skill development, implementation did not go entirely as planned. Initially, all group-coaching sessions were designed to be in person to facilitate community building and open dialogue. Unfortunately, with the pandemic, four sessions were virtual. The content was delivered, but this may have diminished discussion. We also had waning attendance from the faculty participants at the group-coaching sessions. Additionally, there was variation in approach to on-shift coaching. We trained fellows and faculty on a specific approach; however, fellows reported that faculty typically used a more flexible, informal approach throughout the shift.

Although we made efforts to mitigate limitations, these findings should be interpreted in context of the limitations. Our outcomes data used interviews, which relied on recall and may have been influenced by social desirability; we did not measure frequency of skill use. Other factors that were not measured may have contributed to the development of MAL skills. Further, the small sample size at a single institution may limit the generalizability.

### Next Steps

This innovation demonstrates a novel coaching approach to facilitation of MAL development in PEM fellows. Our initial evaluation of this pilot has informed our next steps in enhancing and expanding the program. We identified key factors for success: fellow engagement; group sessions; and faculty participation.

By eliciting input prior to implementation, highlighting need for these skills early, and making skills relevant to the ED, fellows were engaged from the beginning and felt this was relevant to their learning. Going forward, we will include a fellow on our core team to provide ongoing input to ensure continued engagement. We will continue using the group sessions to create an exchange of ideas and normalize the conversation around growth. We are considering ways to harness the expertise of the fellows who completed the initial pilot to teach new fellows while continuing to build their skills throughout their three-year fellowship.

Despite declining attendance, faculty participation in the group sessions demonstrated to the fellows the faculty’s receptivity to having these coaching conversations even when not using the prescribed approach. Fellows felt comfortable seeking input about their development without concern for punitive response. Therefore, we will continue to include faculty in our group sessions and are exploring ways to increase faculty engagement such as limiting faculty sessions, rotating faculty, or seeking compensation.

We identified a gap in our program in facilitating growth in the learning and adjusting phases. This may be attributed to a higher starting point in these areas. However, we are incorporating content on learning strategies in the group sessions and faculty will model searching for resources on shift. For the adjusting phase, fellows have developed an approach to changing their own clinical practice and recognizing when they have mastered this change. We will expand our focus to creating change in the local system by more intentionally linking this process to leadership development and teamwork.

## Figures and Tables

**Table 1:** Overview of coaching approach to facilitate the development of a master adaptive learner.

Activity		Expected change	Metric	
	
Group coaching sessions			Quantitative (Fellow survey) N=6	Qualitative
Session/ MAL phase	Learning objectives			
1. Overview	Describe MAL framework Apply real-time coaching models	Describe MAL framework Apply real-time coaching models	100% (6/6) recognized need for feedback for gap identification 83% (5/6) comfortable seeking feedback 100% (6/6) with specific goals	Planning
2. Planning	Seek out and use feedback Identify gaps on shift Use questioning			
3. Planning	Prioritize gap Set learning goals Search for resources			
4. Planning	Identify gaps outside of clinical shifts			
5. Learning	Identify appropriate resources for each context (eg, on shift vs. reviewing charts) Critically appraise resources Identify active learning opportunities	Use effective approaches to learning	83% (5/6) have standard approach to their learning	Learning
6. Assessing adjusting	Perform informed self-assessment Adjust practice to account for new learning	Use informed self-assessment Incorporate new learning into routine practice		Assessing and adjusting

On-shift coaching		Increased frequency of feedback conversations oriented to development	83% (5/6) regularly had on-shift coaching	Planning
		Increased creation of learning plans for identified gap		
		Develop learning strategies		Learning

Longitudinal coaching		Increased active, iterative learning plans		Planning
		Increased use of active learning plan	83% (5/6) have active plans	Learning
		Fellow engagement in learning process		Overall

*MAL*, master adaptive learner.

**Table 2:** Representative quotes on the impact of program to develop master active learners.

Phase of MAL	Representative quotes
PLANNING Learner identifies gap in practice (knowledge, skill, or attitude), prioritizes learning gap, and then searches for appropriate resources for learning.	*“It is so helpful when an attending tells me what I did wrong and then works with me to identify how I can do better next time…and then helps me figure out an action plan to do better.”* *“[Another fellow] shared last time that he asks the inpatient team for feedback after he admits them to see what he could have done differently - I have started doing that too and have recognized some things to work on.”* *“I have to have some time and space to think about it. So, now I write it [the topic] down after shift or maybe a patient MRN and then return to it the next day when I’m not so tired.”* *“I have actual goals for the big things that we review [with the PD] like working on flow. But for the rest of the stuff, it really depends on what walks in the door, and I address the gaps as they come up.”* *“The meetings [with the PD] help me set expectations for myself for what I should be able to achieve at this level of training and identify how to work towards that.”* *“[The group sessions] were really helpful – now we look out for each other on shift to point out learning opportunities if we know someone is trying [to work on a particular area] “*
LEARNING Learner engages in meaningful learning, appraises resources, and then uses active learning strategies.	*“My approach to this hasn’t changed too much, but I have learned some tips from the others.”* *“I have different strategies depending on where I am. On shift, I look at UptoDate, ask an attending or quick try to find an article. Usually though I am only putting out fires [on shift] and looking up what I have to. And then try to keep a list to look up later. I started doing this early in fellowship, and I don’t think it has really changed [during this pilot].”* *“If I identify something totally out of my knowledge base but I have to consult someone anyways, I don’t worry about really understanding it in that moment if I’m busy. But I’d ask them (the consultant) questions about what I need to know in my role, and maybe I’ll try to follow-up for more later. I started doing this in residency.”*
ASSESSING Learner tries out what they have learned by comparing and contrasting their self-assessment with external feedback	*“The feedback made me realize that I hadn’t improved at talking to families as much as I thought I had. We worked together to make a new plan.”* *“Group sessions with faculty show me they are open to having these conversations, even if they don’t get the words totally right.”* *“All of this primes me to receive feedback and try to compare it to what I thought [about how I did] and then figure out what I don’t know and what to work on.”* *“I try to reflect after shift on what I tried and what I need to learn from there and also about any progress on bigger goals.”*
ADJUSTING Incorporate new learning into practice	*I feel like I have mastered a goal when I am very comfortable and can integrate it into natural workflow and teach about it. For example, when I have a kid with an arm injury if I can read the elbow x-rays and make a plan with the resident before staffing or consulting ortho[pedics], I feel like I’ve mastered it. I’m not sure this has changed much.”* *“I really feel comfortable with something after I do it on shift a few times, but I think this has always been true.”*

*PD*, program director.
